# The role of phenotypic plasticity on the proteome differences between two sympatric marine snail ecotypes adapted to distinct micro-habitats

**DOI:** 10.1186/1471-2148-10-65

**Published:** 2010-03-08

**Authors:** Mónica Martínez-Fernández, María Páez de la Cadena, Emilio Rolán-Alvarez

**Affiliations:** 1 Departamento de Bioquímica, Genética e Inmunología, Facultad de Biología, Universidad de Vigo, Campus Universitario, 36310 Vigo, Spain

## Abstract

**Background:**

The role of phenotypic plasticity is increasingly being recognized in the field of evolutionary studies. In this paper we look at the role of genetic determination *versus *plastic response by comparing the protein expression profiles between two sympatric ecotypes adapted to different shore levels and habitats using two-dimensional protein maps.

**Results:**

We compared qualitative and quantitative differences in protein expression between pools of both ecotypes from different environments (field and laboratory conditions). The results suggested that ecotype differences may affect about 7% of the proteome in agreement with previous studies, and moreover these differences are basically insensitive to environmental changes. Thus, observed differences between wild ecotypes can be mainly attributed to genetic factors rather than phenotypic plasticity.

**Conclusions:**

These results confirm the mechanism of adaptation already proposed in this species and a minor role of phenotypic plasticity in this ecological speciation process. In addition, this study provides a number of interesting protein spots potentially involved in adaptation, and therefore candidates for a future identification.

## Background

The ability of populations to adapt to a particular habitat is a key topic in evolutionary biology because the exploitation of new niches is a key component of the speciation process [1]. In order to survive in heterogeneous habitats, organisms can adopt three main evolutionary strategies [2]. One is to fix a generalist genotype more or less suitable over a broad range of environmental conditions. A second possibility is to adapt to a particular environmental condition by heritable variation in a particular trait (direct genetic determination). Finally, a third option is to (genetically) control the sensitivity of the genotype to changes in the environment (i.e. indirect genetic determination; phenotypic plasticity). The first and third strategies are expected to be typical of organisms with relatively high dispersal abilities, while the second strategy is preferentially expected for species with restricted dispersal ability, although different exceptions are known [3]. In addition, in the case of sedentary organisms the level of environmental fluctuation might also affect which strategy is used [4,5].

Phenotypic plasticity is defined as occurring when the phenotype expressed by a given genotype is altered by changes in environmental conditions [6]. Therefore, it is possible to quantify the relative importance of direct genetic determination *versus *phenotypic plasticity for causing adaptative variation in a particular trait, even though the plastic ability of the trait can show heritable variation within and between populations and species [7,8]. A body of evidence suggests that plasticity may promote adaptative divergence in various systems, often followed by genetic changes in the direction of the plastic response [9]. Furthermore, phenotypic plasticity enhances the survival and reproductive success of individuals by contributing to their ability to cope with environmental changes. In this way, it enables of potential adaptation to new niches [10], and therefore can promote important biological processes such as adaptation, divergence and reproductive isolation. However, we cannot, *a priori*, assume that phenotypic plasticity is always an adaptative response under natural selection [11]. When studying the mechanism (genetic or plastic) of adaptation, it may help to identify a clear model system in which phenotypic variation has been shown to be clearly adaptative. Here, we use two well-known ecological forms (ecotypes) of a marine snail as a biological model system in order to study the role of phenotypic plasticity in potentially adaptative traits.

*Littorina saxatilis *(Olivi) is a marine intertidal gastropod that presents separate sexes, ovoviviparity (in which females carry a brood pouch with non-planktonic shelled embryos) and high polymorphism. Along of the Galician coast (NW Spain), two intertidal ecotypes of the snail *L. saxatilis *are adapted to different shore levels and habitats [12,13]. The large-sized ridged and banded ecotype (RB), lives among barnacles at the upper shore, has a larger, thicker and more robust shell, and a smaller aperture for reducing the loss of water due to the high desiccation from exposure to sunshine [14-16]. At the lower shore, the small-sized, smooth and unbanded ecotype (SU) lives on mussels, and has a smaller and thinner shell with a relatively wider aperture necessary to accommodate a larger muscular foot that prevents dislodgement resulting from heavy wave action [14-16]. Both habitats and ecotypes are typically separated by 5-10 meters, although the snails have the potential ability to move from one habitat to the other during their lifetimes, and hence can be considered as effectively sympatric [13]. At the mid-shore, both ecotypes meet and occasionally mate in true sympatry, showing a partial pre-zygotic isolation barrier (i.e. mating assortatively; [13]). Due to the effectively sympatric ecotype distribution and its incomplete reproductive isolation, the gene flow among ecotypes is only slightly restricted [13,17]. Therefore, the polymorphism observed is due to a strong, divergent natural selection acting across the environmental gradient [14,16,17], making a perfect system to study the genes involved in the origin of adaptation and speciation processes.

These ecologically distinct ecotypes have shown differences in about 3% of their genome that can not be explained by stochastic forces, reported as the first preliminary estimate of the percentage of the genome variation affected (directly or indirectly) by natural selection in this species [17]. In addition, a few studies in the same species showed that shell shape variation is an adaptive trait along the vertical rocky shore gradient, although its phenotype is relatively independent of the experienced environment [16,18]. In fact, the majority of the adaptative morphological variation was attributed to direct genetic determination (fixed differences between ecotypes; [18]). Recently the proteome profiles of these two ecotypes have been compared using two-dimensional electrophoresis [19]. In such study, the two sympatric ecotypes collected in the field differed in 12% of their proteome, 7.2% after correcting for multiple testing [reanalyzed in [17]]. In the former study, however, the observed differences between ecotypes could be explained by both genetic and environmental determination, since protein expression profiles are known to be significantly affected by environmental changes [20,21]. Here, a new proteome comparison between field-collected and laboratory-reared snails of both RB and SU ecotypes was carried out to study the possible role of phenotypic plasticity in determining the protein expression in this model system.

## Results and Discussion

### Analyses of Two-dimensional Protein Profiles

**Figure 1:**
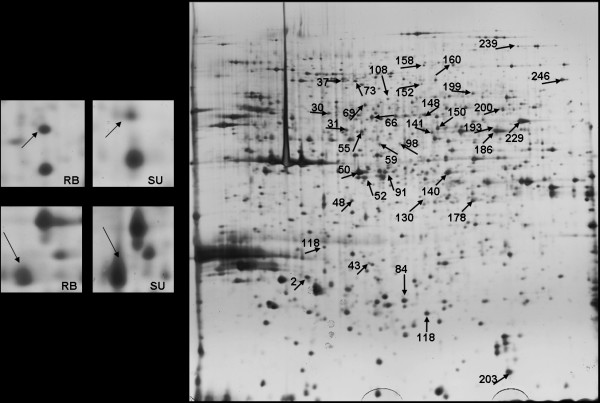
**Example of Two-dimensional Protein Map**. Two examples of spots showing significant differentiation between ecotypes (spot 48 and 200) are shown for one specimen of each ecotype (left). Two-dimensional protein map from an RB specimen (right). Spots with altered expression are indicated by arrows, and numbered as in Table 2.

Protein expression profiles of pooled snails from different ecotypes (RB and SU) and habitats (field and laboratory) were studied. After 2-DE (two-dimensional gel electrophoresis), only well-resolved protein spots were taken into account for the analyses, finding 446 spots on average per gel. In the qualitative analysis, 764 spots altogether were observed in at least one of the 12 gels, finding 7 significant spots (0.9%) between ecotypes after multitest adjustment, while none remained significant between environments (Table 1). In the quantitative analysis, only 247 spots were present in all gels, obtaining 17 spots (6.9%) with significantly different expression between ecotypes after multitest correction, and none between environments (Figure 1). This estimate was nearly identical to the estimate obtained by Martínez-Fernández *et al. *(2008) [19] comparing the same ecotypes from the same locality, pointing to the robustness of the proteomic approach (Table 1). This similarity between the different estimates is notable as the two studies used specimens of different ages (juveniles *versus *adults), years and seasons. Only two (spot numbers 84 and 160) out of the 247 spots studied did not show homocedasticity, but they remained significant after being transformed, which supports the assertion that the former pattern is statistically robust.

**Table 1:** Summary of the results obtained in the qualitative and quantitative comparisons of protein expression between *Ecotype *and *Environmental *factors.

	Source	N spots	% Ecotype	% Environment
**Qualitative**	2008 data^1^	1498	0.8% (1.4%)	-
	Present data	764	0.9% (1.7%)	0% (0.5%)
			
	Mean ± SE		0.85% ± 0.05	

**Quantitative**	2008 data^1^	136	7.4% (16.2%)	-
	Present data	247	6.9% (14.6%)	0% (5.3%)
			
	Mean ± SE		7.1% ± 0.25	

1 Data re-analysed from Martínez-Fernández et al. (2008) [19]

*N spots *is the number of spots studied, *% *from *Ecotype *or *Environment *is the percentage of spots, respectively, being statistically different between treatments within factor. The numbers in parenthesis are the percentages with significant differences in expression before multitest correction. The mean value ± standard error (SE) from the percentage of differences due to ecotype is represented as well.

### Quantifying the Importance of Phenotypic Plasticity

A detailed two-way ANOVA was carried out in those protein spots that showed significant differentiation for any factor separately. This analysis included *Ecotype*, *Environment *and *Interaction *factors, and allowed us to estimate the relative importance of each one using eta squared (η^2^) coefficients (Table 2). The results confirmed that the *Ecotype *is the main factor, explaining on average 73% (range 40-98 among different spots) of the overall variance in expression, whereas the *Environment *explained an average of 18% (range 0.6-43), and the *Interaction *an average of 8% (range 0-21.88%). In fact, these mean percentages across factors differed significantly (Table 1). The low level of genotype-environment interaction has been interpreted in similar studies as a preference for additive effects controlling gene expression [22], although epistatic (non-additive) effects were observed at least in one study using microarrays [23]. Furthermore, if plasticity is contributing to adaptation, the differences in expression between ecotypes will be higher in the field than in the laboratory environment. In this study, only 2 of 17 (12%) of the proteins with differences in expression between ecotypes showed also significant differences between environments in the two-way ANOVA. Moreover, only in one of these cases (spot 193) plasticity could contribute to maintain this polymorphism, since the different expression between ecotypes is still higher in the field than in the laboratory (Table 2). But, even in that case, the percentage of variation attributed to the environment (35%; see Table 2) was smaller compared to the percentage attributed to the ecotype differences (43%).

**Table 2:** Levels of Expression for Each Protein Spot Showing Differences in Expression.

Spot	RB	SU	Ratio	Ecotype	Environment	Interaction
30	724.64 ± 103.78	216.71 ± 100.13	3.34	79.25**	12.05	8.7
43	70.59 ± 43.72	386.98 ± 81.09	-5.48	95.79*	4.03	0.18
48	378.32 ± 87.32	68.08 ± 41.28	5.56	62.36**	27.92	9.72
50	1674.41 ± 379.13	681.51 ± 204.32	2.46	40.39***	43.5***	16.11
52	1865.46 ± 376.09	443.32 ± 237.86	4.2	80.09*	5.35	14.56
59	361.68 ± 53.20	151.07 ± 49.41	2.39	72.43*	22.71	4.86
66	1583.49 ± 280.381	670.42 ± 152.39	2.36	65.79*	32.21	1.99
84	1232.97 ± 240.93	2939.03 ± 526.56	-2.38	71.70*	28.3	0
98	72.73 ± 37.19	296.12 ± 56.54	-4.07	87.64*	1.62	10.84
148	829.97 ± 77.12	356.78 ± 72.94	2.33	98.38**	0.59	1.03
150	1389.79 ± 158.25	787.20 ± 167.70	1.76	54.23*	34.23	11.54
158	258.26 ± 45.66	91.68 ± 36.10	2.82	71*	20.93	8.06
160	492.35 ± 69.44	105.69 ± 89.84	4.66	61.86**	34.72	3.42
193	79.15 ± 47.43	505.40 ± 146.02	-6.39	43.42**	36.71**	19.87
200	625.99 ± 161.99	1395.59 ± 204. 96	-2.23	75.59*	2.53	21.88
203	2034.47 ± 374.81	809.62 ± 103.50	2.51	98.23*	1.77	0
239	488.00 ± 98.69	40.73 ± 22.97	11.98	89.88**	5.29	4.9
				
			MEAN	73.41	18.49	8.09
			*P*_randomization_		0.0001	

*P ≤ 0.05; **P ≤ 0.01; ***P ≤ 0.001

Mean levels of expression in ppm (± standard errors) for significant spots in the one-way ANOVA. The Ratio quantifies the expression differences between ecotypes (larger intensity/smaller intensity) with the sign showing the ecotype with larger intensity (+ for RB and - for SU). Ecotype, Environment and Interaction represent the percentage of variance explained after the two-way ANOVA (the relative importance of the η^2 ^in percentages). *P *is the significance of the randomization ANOVA comparing percentages of variation across factors (see [52]).

There is a few possible sources of biases that could potentially affect to our experimental design. For example, we can not exclude that some juveniles were fertilized in the wild, since the females were maintained in the lab during 2-4 months and the time that zygotes need to develop into juveniles inside the mother is still unknown. However, females have a sperm reserve that can store for months (even more than 6 months; [13]) and we already proved that after three months in laboratory females nearly finish releasing new born juveniles [24]. Thus, we expect that most born juveniles were developed in the lab, if not also fertilized, thanks to this sperm reserve. Additionally, since we did not maintain our snails for two generations in laboratory conditions, we can not neglect completely a contribution of maternal effects to our genetic factor. Nevertheless, maternal effects seem to be typically genetic in origin [25], and therefore, we did not expect that the main picture outlined here would change considerably. Such conclusion is particularly robust when thinking in a trait like protein expression, which considerably varies spatially and temporally [21].

In summary, all these results support a relatively minor contribution of the phenotypic plasticity to the ecotype differences observed in the proteome expression, suggesting an important genetic basis for the variability in gene expression in this species. Future studies should focus on the estimation of the heritability in protein expression profiles.

A comparison of the same causal factors was carried out in the same species and population for shell shape variation [18], indicating that most shell shape variation was also accounted by the ecotype of the parents, irrespectively of the experienced environment. The similar relative importance of genetic effects in morphology (range 72.7-97.3%) and protein expression (on average 73.4%; see Table 2) points to the generality of the phenomenon, since the mechanisms that enable the plastic responses at the different biological levels are fundamentally the same [26]. Therefore, this species seems to show low levels of phenotypic plasticity affecting variation in morphology [18] and proteomic expression (this study).

A few studies have observed proteome adaptation in plants [22,27], where two populations of *Picea abies *showed different expression profiles at two different ecological habitats. In addition, proteome expression variability associated with particular taxa [28,29], or with populations living in different environments [30,31] or affected by distinct pollutants [32,33] has been observed in several marine bivalve molluscs. However, in spite of the general importance of phenotypic plasticity in adaptation, to our knowledge there has not been any study focusing on the quantitative relevance of phenotypic plasticity on protein expression, making evident the importance and novelty of this work. In fact, most of the studies focused on the role of phenotypic plasticity have been traditionally carried out on the basis of the morphological differences [10,34,35], although some used DNA markers [36] or new large-scale gene expression technologies [reviewed in [37]]. Even when these studies have found a possible role of plasticity in different biological processes, neither of them quantified the relative role of genotype *versus *environment effects.

### Cluster Analysis

**Figure 2:**
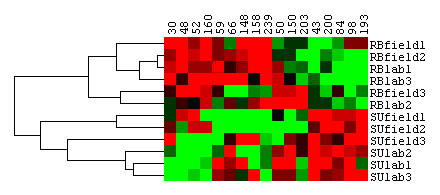
**Cluster Analysis**. Cluster analysis on the 17 protein spots with significantly different expression between ecotypes after multitest correction. Rows represent the pooled individuals from each ecotype and environment, and columns represent the protein spots. Red indicates enhanced expression while green reflects decreased expression. The pools were correctly clustered by ecotype based on their protein profiles.

A cluster of the 17 protein spots showing quantitative differences in expression was applied in order to detect groups of spots or specimens with similar expression levels. The individuals were correctly clustered by their ecotype. In addition, the protein spots were clearly grouped in two well-differentiated classes (Figure 2). Such clustering has been traditionally interpreted as protein co-regulation, which may indicate that they are participating in similar metabolic pathways [38,39]. Therefore, future efforts will need to focus on the identification of these spots in order to confirm or reject this hypothesis. Moreover, it could be interesting to study them at different developmental stages in order to characterise the biochemical and physiological strategies of adaptation, especially after knowing that they showed genetically controlled expression differences between ecotypes.

## Conclusions

To conclude, understanding how biological diversity is generated and maintained is a central issue in evolutionary biology. We have detected that a portion of the proteome studied (about 7%) differs in expression level between ecotypes, possibly related with their adaptation to the distinct habitats and shore levels. In addition, phenotypic plasticity does not have an important role in determining those proteomic differences, supporting the assertion that most of the proteomic variation may have a genetic origin, at least in those with differences between ecotypes. However, this information is still insufficient to reveal the genetic architecture of the adaptative phenotypic change, since to do this purpose it would be necessary to identify quantitative trait loci of gene expression profiles, as well as their genetic architecture (being coding or non-coding regions), an extremely difficult but interesting future task for this evolutionary model system.

## Methods

### Sample Collection and Preparation

In November 2007, wild females of *L. saxatilis *were collected in Silleiro (NW Spain) and taken to the laboratory where they were placed in an aquarium. Females of the RB ecotype were obtained from the upper shore, whereas the females of the SU ecotype were obtained from the lower shore. In the laboratory, a continuous sea water flow at 14.2°C, 3.63% of salinity, and an oxygen level of 7.6 mg/L was maintained by an open circuit. The system was also provided with a 14/10 h photoperiod (daylight/darkness) supplied through fluorescent lighting. Further technical details of the breeding system in this species are given in previous works [16,18]. The pregnant females were maintained in the laboratory 2-4 months before the analysis of recently born juveniles, to minimize the possible contribution of maternal effects to the experimental specimens. In June 2008, around 40 specimens in the juvenile stage (3-6 months old) were frozen at -80°C. The day after, a similar number of juveniles (similarly sized) were collected from Silleiro, at the same site where the mothers of the laboratory-reared juveniles were captured, and immediately frozen as well at -80°C. For each combination of ecotype (RB and SU), and environment (field- and laboratory-reared), we prepared 3 samples, each one including a pool of 10 individuals in order to discard individual differences as described in [19]. This design allows us to compare differences in gene expression between ecotypes experiencing drastically different environments.

Shells were removed and tissues were homogenised in lysis buffer [7 M urea, 2 M thiourea and 4% (w/v) CHAPS] with protease inhibitors (Complete Mini, Roche) to a final ratio of 50 mg tissue per 1 mL of lysis buffer. The homogenates were stored at -80°C until they were further analysed. Then, proteins were solubilised at 100 rpm and 25°C for 1 h in an orbital shaker, and finally centrifuged at 16,000 g for 15 min. Supernatants were immediately used for electrophoresis, and protein concentration measured according to Bradford (1976) [40] with modifications [41].

### Two-dimensional Gel Electrophoresis, Image Acquisition and Spot Detection

Two-dimensional gel electrophoresis (2-DE) is one of the most widely used techniques for separating complex biological mixtures containing large numbers of proteins, and remains one of the key methodologies in proteomic studies [21]. This comprehensive technology uses two sequential electrophoretic runs to separate the proteins in a particular sample. In the first dimension, the proteins are separated on basis of their isoelectric point (p*I*) (through an immobilized pH gradient), using strips with a pH ranging from 5 to 8, and loading 150 μg of protein per gel. In the second dimension, the proteins already separated by their charge are separated in 12%-polyacrylamide gels depending on their relative molecular mass (M_r_) (through a porosity gradient). The 12 replicates, 3 for each combination of ecotype and environment, were run in a pseudo-random sequence to randomize uncontrolled technical/laboratory factors. After 2-DE, gels were stained with silver nitrate [42] but with modifications in order to be compatible with mass spectrometry, obtaining a protein map from each sample. Finally, the normalized volume for each protein spot was compared for all 12 maps. Further technical details of 2-DE and the image analysis are explained in Martínez-Fernández et al., 2008 [19]. A qualitative analysis was performed using all protein spots detected with their intensities transformed into a matrix of 0 (absence) and 1 (presence). The quantitative analysis was carried out only on those spots present in all the 12 replicates studied, using their relative spot volumes (quantities) normalised to the full spot intensity of each gel (see [19]), actually representing a semi-quantitative analysis [30,43-45]. Note that, in the qualitative analysis, the absence of a particular spot in a particular gel does not guarantee absence of expression, rather that its expression could not be detected by the technique. However, qualitative and quantitative analyses allow us to investigate a larger number of spots in expression profiles by different statistical methods.

### Statistical analyses

First, we compared the qualitative and quantitative spot incidence in the ecotype (RB and SU) and environment (field and laboratory) factors separately. The comparison of the presence/absence of detected spots across the 6 replicates of the two factors was accomplished by a Fisher contingency exact test. In order to avoid high rates of false positives we used a significance level of 0.2% (a probability of being caused by chance of 0.002, i.e. representing spots present in all replicates of one ecotype and none of the other) [19].

In the case of the quantitative analysis, we first used the Levene test to check if the relative spot intensity showed homoscedasticity in order to compare mean differences in spot intensity between factor treatments using a one-way ANOVA. Moreover, although deviations from normality are of less importance under ANOVA [46-48], none of the protein spot residuals analysed deviated from normality under the Kolmogorov-Smirnov test. In the two cases where the homoscedasticity was not met, they were transformed using logarithm and square root. The significance of both qualitative (adjusted to 0.2% with multiple testing), and quantitative analyses (adjusted to 5%) was corrected for multiple testing using the SGoF correction. This statistical method has been shown to present the highest statistical power when the number of tests is high and the sample size low, without increasing appreciably the rate of false discoveries compared to other alternatives [49]. The SGoF software can be freely obtained at http://webs.uvigo.es/acraaj/SGoF.htm.

In addition, for those protein spots showing quantitative differences in expression, we conducted a two-way ANOVA to estimate the relative importance of the factors *Ecotype *and *Environment *and their *Interaction *following the method reported in Scheiner (1993) [50]. On the one hand, the factor *Ecotype *(fixed; RB and SU) allowed us to estimate the genetic contribution effects (perhaps partially biased by environmental differences in the wild). In this sense, if two populations/families maintain their phenotypic differences irrespective of their environment, it can be taken as evidence of genetic determination [25], and therefore the results of this experiment could suggest *a posteriori *that ecotype differences might be caused mostly by genetic factors. On the other hand, the factor *Environment *(fixed; field and laboratory-reared) accounts exclusively for environmental effects, whereas the *Interaction *represents possible genetic-environment interactions [18]. Moreover, the relevance of the factor *Environment *will show the possible contribution of phenotypic plasticity to this polymorphism. Note that even if the ecotype differences are affected by the environment, only those cases in which the magnitude of the ecotype differences increases more notably in the field than in the laboratory are expected to favour the maintenance of this polymorphism [18].

The relative magnitude of a fixed effect (or interaction) can be directly estimated by the eta squared (η^2^), which gives the percentage of variation explained by each factor in the ANOVA [48,51]. We present the magnitude of the η^2 ^of each factor as a percentage of the sum of the three factors. Therefore, with this analysis we could investigate to what extent the presumed adaptative proteome differentiation was influenced by genetic *versus *plastic factors.

Additionally, a hierarchical clustering was applied to the protein spots showing quantitative patterns of expression using the Euclidean distance and the average linkage algorithm by the Cluster 3.0 and Java Treeview software. All other statistical analyses were accomplished with the SPSS/PC software ver. 16.0.

## Abbreviations

IAA: iodoacetamide; RB: ridged and banded; SDS-PAGE: denaturing polyacrylamide gel electrophoresis; SU: smooth and unbanded.

## Authors' contributions

Authors' contributions: MMF: AB & ES, MPC: FG, ERA: ES & FG. All authors read and approved the final manuscript.
